# Supplementary material for: Maternal High-Fat Diet Induces Sex- and Estrous Cycle–Specific Glial Dysregulation in the Peripheral Offspring Retina
Source: Invest Ophthalmol Vis Sci. 2026 Aug 7;67(10):20. doi: 10.1167/iovs.67.10.20 (PMC13456062; doi:10.1167/iovs.67.10.20)
Supplement: Supplementary file 1 [file iovs-67-10-20_s001.pdf]

Supplementary Material

Maternal High-Fat Diet Induces Sex- and Estrous Cycle –Specific Glial Dysregulation in the Peripheral Offspring Retina

Gintare Urbonaite[1], Patricija Cepauskyte[1], Neda I. Biliute[1,2], Guoda Laurinaviciute[3], Daiva Dabkeviciene[1], Solveiga Samulėnaitė[1,4], Urte Neniskyte[1,5]

[1] Institute of Biosciences, Vilnius University Life Sciences Center, Vilnius, Lithuania
[2] Laboratory of Cognitive and Adaptive Neurosciences (LNCA), University of Strasbourg, Strasbourg, France
[3] Institute of Biomedical Sciences, Faculty of Medicine, Vilnius University, Vilnius, Lithuania
[4] Department of Biological Models, Institute of Biochemistry, Vilnius University Life Sciences Center, Vilnius, Lithuania
[5] VU LSC-EMBL Partnership Institute for Genome Editing Technologies, Vilnius University Life Sciences Center, Vilnius, Lithuania

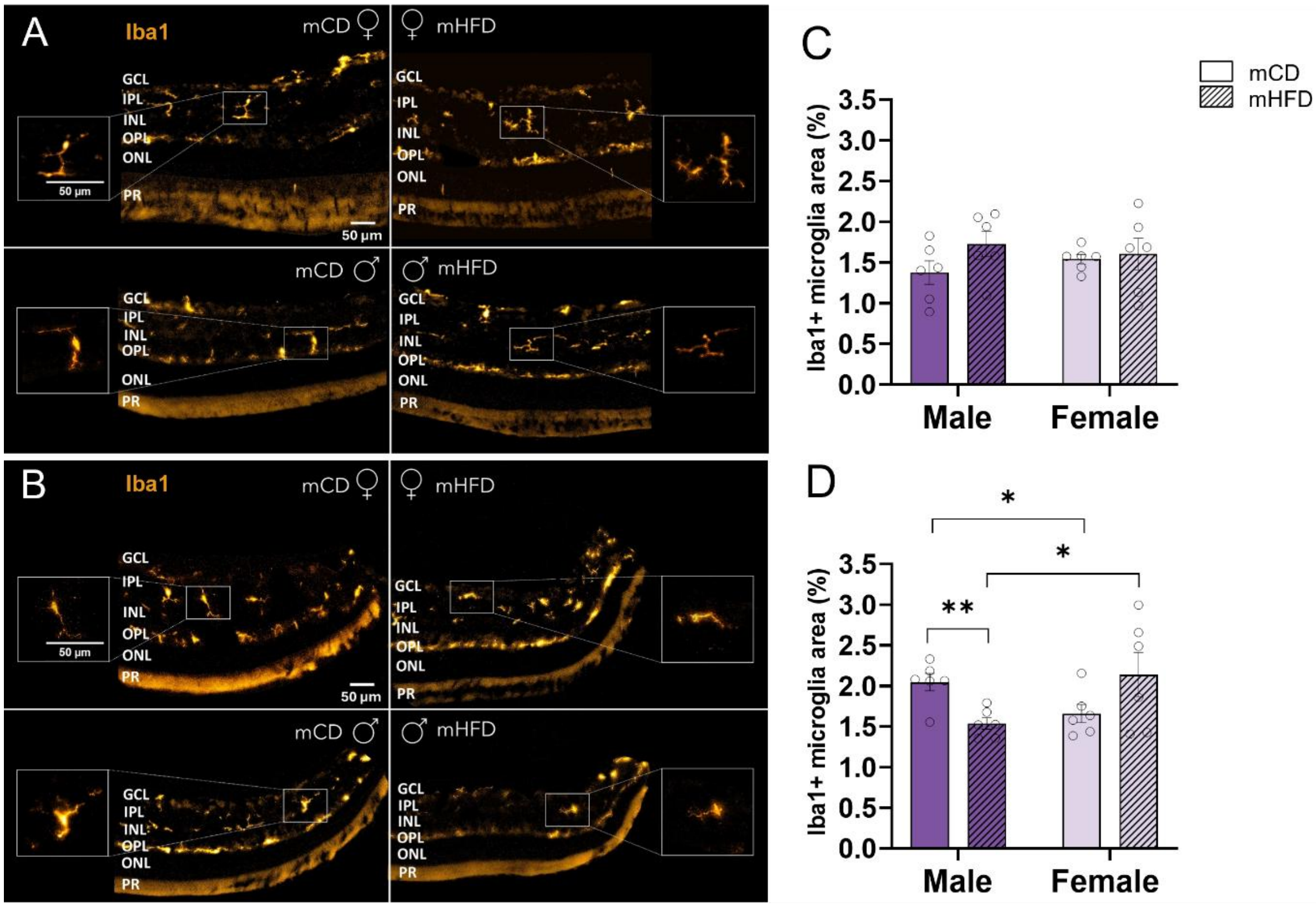

Supplementary Figure 1. The effects of mHFD on the Iba1-marked microglia of the offspring retina. Representative confocal images of Iba1-positive microglia in central retina (A) and peripheral retina (B) sections from female (♀) and male (♂) offspring of dams who were fed either a control diet (mCD) or a high-fat diet (mHFD). (C) Iba1+ microglia area (µm²/mm²) in the central retina of the offspring. (D) Iba1+ microglia area (µm²/mm²) in the peripheral retina of the offspring. Mean ± SEM, (n = 6 animals per group). *P* values were assessed using nested two-way ANOVA with Tukey's post hoc tests. $^{*}P < 0.05$, $^{**}P < 0.01$, $^{***}P < 0.001$, $^{****}P < 0.0001$.

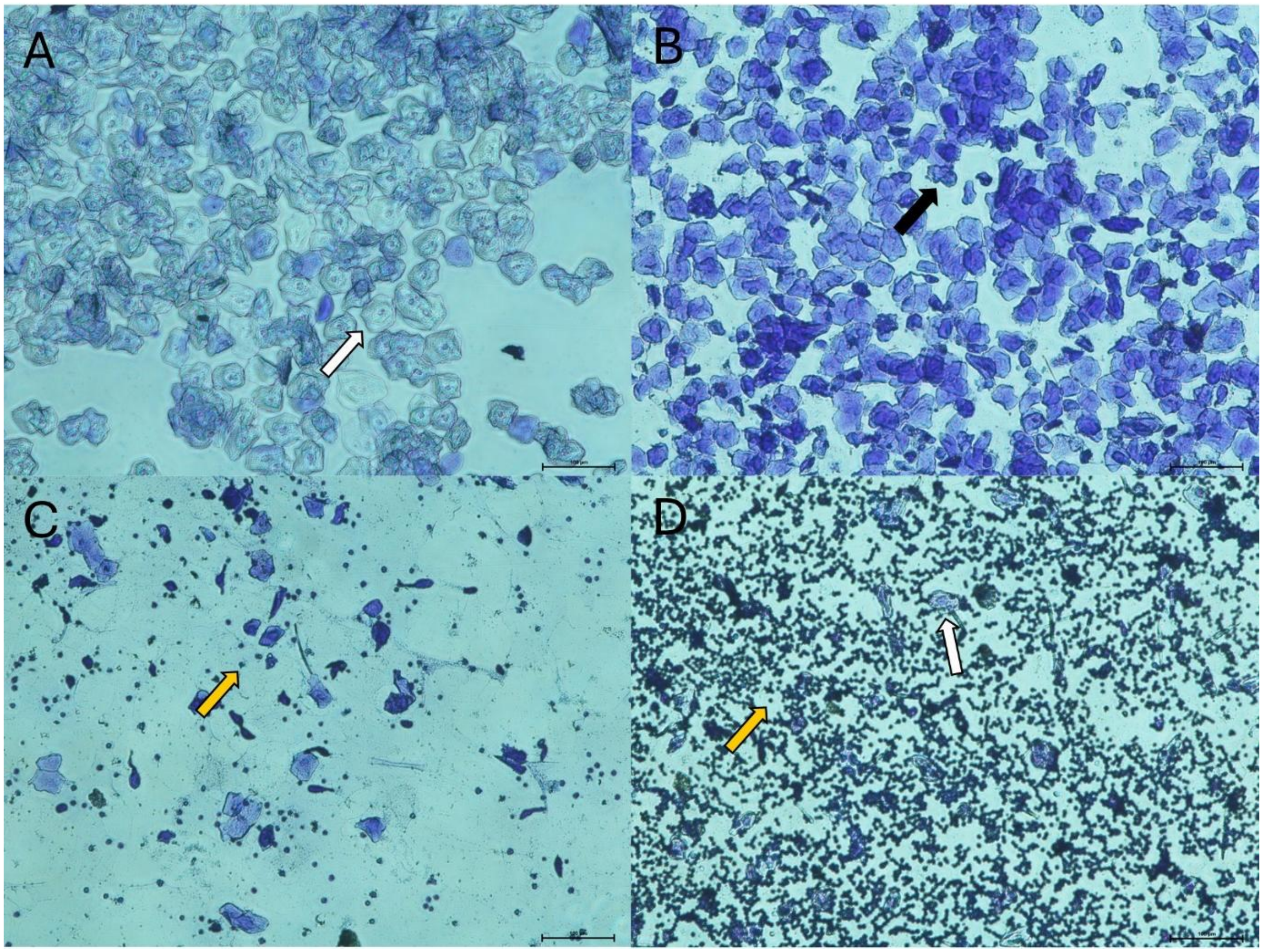

Supplementary Figure 2. Estrus cycle phase determination from vaginal smears stained with crystal violet. (A) The proestrus phase is characterized by the predominance of nucleated epithelial cells (white arrow). (B) The estrus phase is defined by the predominance of anucleated, cornified, irregularly shaped epithelial cells (black arrow). (C) Metestrus phase is identified by predominance of leukocytes (yellow arrow). (D) Diestrus phase is determined by the presence of both leukocytes (yellow arrow) and some nucleated epithelial cells (white arrow). Scale bar: 100 µm.

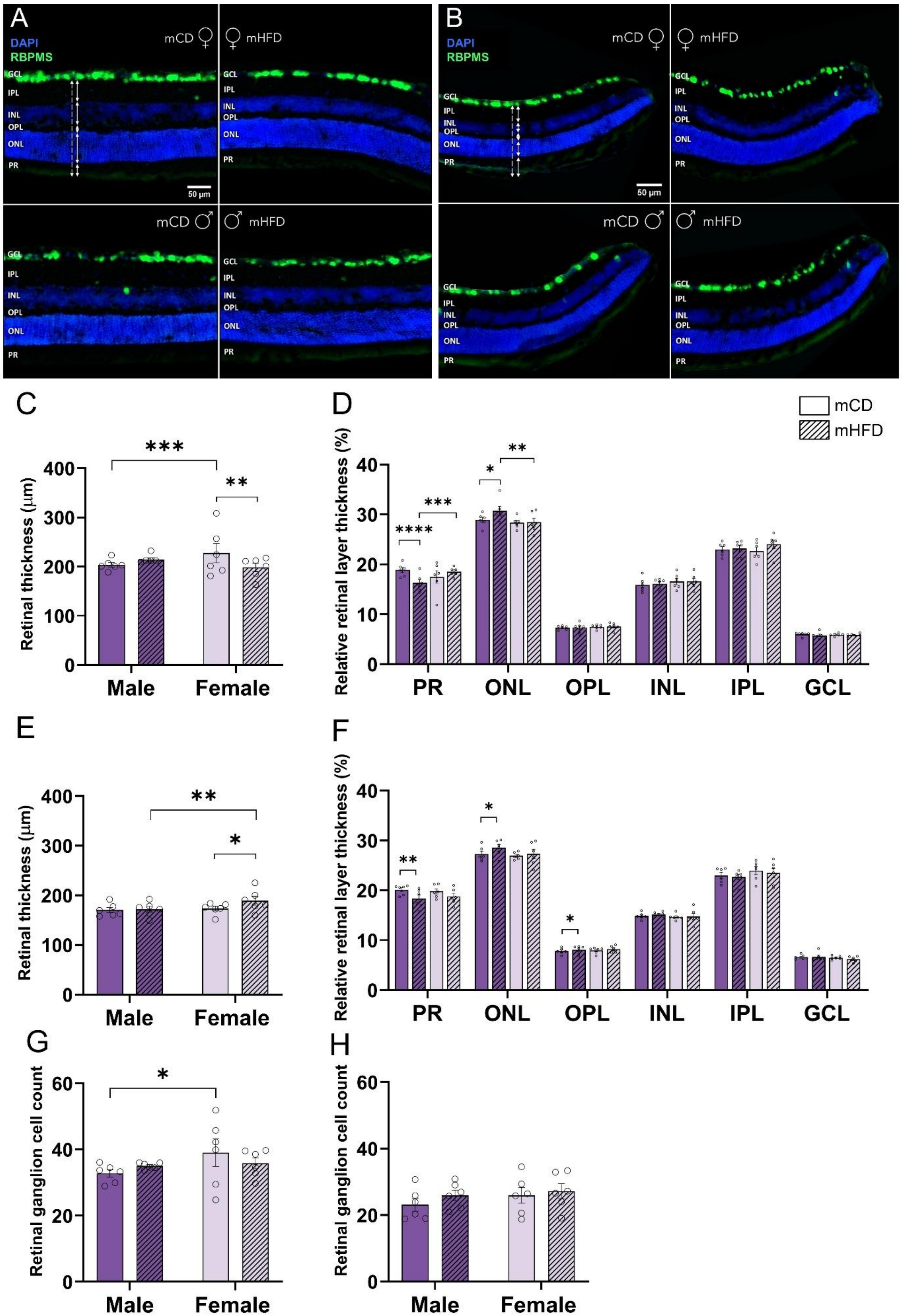
A
DAPI
RBPMS
mCD ♀
♀ mHFD
mCD ♂
♂ mHFD
GCL
IPL
INL
OPL
ONL
PR
50 µm
B
C
Retinal thickness (µm)
Male
Female
D
Relative retinal layer thickness (%)
mCD
mHFD
PR
ONL
OPL
INL
IPL
GCL
E
F
G
Retinal ganglion cell count
H

Supplementary Figure 3. The effects of mHFD on the offspring's retinal thickness and ganglion cell density. Representative confocal images of RBPMS-labeled (green) retinal sections of central (A) and peripheral (B) retina from female (♀) and male (♂) offspring of dams who were fed either a control diet (mCD) or a high-fat diet (mHFD). GCL, ganglion cell layer; IPL, inner plexiform layer; INL, inner nuclear layer; OPL, outer plexiform layer; ONL, outer nuclear layer; PR, photoreceptor layer. Scale bar: 50 µm. (C, D) Retinal thickness (µm) and relative retinal layer thickness (%) in the central retina of the offspring. (E, F) Retinal thickness (µm) and relative retinal layer thickness (%) in the peripheral retina of the offspring. (G, H) Retinal ganglion cell number per 500 µm in the central and peripheral retina of the offspring. Mean ± SEM, (n = 6 animals per group). *P* values were assessed using nested two-way ANOVA with Tukey's post hoc tests. **P* < 0.05, ***P* < 0.01, ****P* < 0.001, *****P* < 0.0001.
